# Supplementary material for: Natural salivary gland barrier curtails Zika virus transmission in Thai Aedes aegypti
Source: PLoS Negl Trop Dis. 2025 Oct 28;19(10):e0013451. doi: 10.1371/journal.pntd.0013451 (PMC12578335; doi:10.1371/journal.pntd.0013451)
Supplement: Attachment — Submitted filename: Sartsanga PLoSNTD 2025.docx [file pntd.0013451.s001.docx]

This is a very well written manuscript comparing the transmission potential of different Aedes aegypti populations from Thailand. They performed numerous assays to examine the observed differences in ZIKV titers in the saliva between the populations including ZIKV infection distribution in salivary glands (BTW the microscopy was really nice), measuring saliva quantity, and the potential role of virus inactivation by the saliva. They also did some experiments to examine the male mating competitive between populations and fecundity. Overall, the experiments were well executed; however, I did have concerns about some of the transmission assays, their analysis and the resulting conclusions. Consequently, I feel that the premise of the manuscript is fundamentally flawed. I’ll try my best to explain my logic below.

The authors include the virus negative saliva samples in their transmission analysis; however, doing so convolutes the interpretation. When performing infection studies in mosquitoes there are two pieces of data that can be collected; how many individuals become infected (prevalence) and what is the burden of infection in those that became infected (intensity) (in this specific scenario virus in saliva and salivary glands). These are two fundamentally different questions and, therefore, should be separated in the analysis in my opinion. Looking at the data presented in Fig 1, it appears that NAK and CSP have lower levels of virus in the saliva than the DMSC strain but upon removal of the negative saliva samples this difference, especially for CSP, most likely disappears. Because so few had infection in the NAK strain it is difficult to determine if there would be a difference. Regardless, does the titer in the saliva actually matter? A previous study found that the human ID50 <10 pfu for dengue virus vaccine candidates (Blaney  doi: [10.1007/978-3-642-02215-9_11](https://doi.org/10.1007/978-3-642-02215-9_11)). To me, the more important metric is the prevalence of ZIKV in the saliva.

With that said, there are problems with the approach for assaying transmission, specifically the use of capillary tubes. A recent study (Gloria-Soria 2022 “Saliva collection via capillary method may underestimate arboviral transmission by mosquitoes”) demonstrated that “the methods of detecting virus infection in mosquito legs and in SG were equally accurate in predicting transmission. Overall, the presence of virus in mosquito legs was a more accurate predictor of transmission than the commonly implemented viral detection method using forced salivation into a capillary tube, and was subject to less technical variation.” Further, what is being collected during forced salivation may not accurately represent what is being salivated during blood feeding. Marinotti et al (“Diet and salivation in female Aedes aegypti mosquitoes”) found that Aae contain alpha-glucosidase in their proximal lobes and apyrase in their distal lobes and when fed a liquid sugar meal, levels of alpha-glucosidase decrease but not that of apyrase while the activity of both enzymes were reduced upon a blood meal. This suggests that the fundamental mechanisms mediating salivation may be different between forced salivation and actual blood feeding. Together, this suggests that the saliva data may not be a good metric to use when assessing transmission potential and that the salivary gland infection data may be better to identify differences between the populations; however, there were no differences at 14 and 21 dpi. Consequently, the premise for these studies and resultant conclusion about salivary gland escape barriers are flawed.

Figure 1: the authors state that “data was summarized from three blood feeding experiments with at least 10 mosquitoes from each”; however, when examining the prevalence data I see only two which I assume represent the replicate experiments. If this assumption is accurate shouldn’t there be three data points for the prevalence data?

Figure 1: It would be nice to include sample size values to the prevalence data in the figure as was done in Fig. 6B.
