# Supplementary material for: Natural salivary gland barrier curtails Zika virus transmission in Thai Aedes aegypti
Source: PLoS Negl Trop Dis. 2025 Oct 28;19(10):e0013451. doi: 10.1371/journal.pntd.0013451 (PMC12578335; doi:10.1371/journal.pntd.0013451)
Supplement: Attachment — Submitted filename: PNTD-D-25-01370 Responses to reviewers resubmission.docx [file pntd.0013451.s003.docx]

**Responses to reviewers PNTD-D-25-01370**

We greatly appreciate the time and effort taken by you and the reviewers to suggest improvements to our manuscript. We have incorporated changes to address the detailed suggestions you have provided. Please see below for the point-by-point responses to comments and concerns raised by the reviewers (in blue).

**Key Review Criteria Required for Acceptance?**

As you describe the new analyses required for acceptance, please consider the following:

**Methods:**
Reviewer #1: See attached comments.

Reviewer #2: Please see my general comments.

Reviewer #3: (No Response)

**Results:**

Reviewer #1: See attached comments.

Reviewer #2: Please see my general comments.

Reviewer #3: (No Response)

**Conclusions:**

Reviewer #1: See attached comments.

Reviewer #2: Please see my general comments.

Reviewer #3: (No Response)

**Editorial and Data Presentation Modifications?**

Reviewer #1: (No Response)

Reviewer #2: Please see my general comments.

Reviewer #3: (No Response)

**Summary and General Comments:**

Reviewer #1: Comments from attached comments.

This is a very well written manuscript comparing the transmission potential of different Aedes aegypti populations from Thailand. They performed numerous assays to examine the observed differences in ZIKV titers in the saliva between the populations including ZIKV infection distribution in salivary glands (BTW the microscopy was really nice), measuring saliva quantity, and the potential role of virus inactivation by the saliva. They also did some experiments to examine the male mating competitive between populations and fecundity. Overall, the experiments were well executed; however, I did have concerns about some of the transmission assays, their analysis and the resulting conclusions. Consequently, I feel that the premise of the manuscript is fundamentally flawed. I’ll try my best to explain my logic below.

**Response:** We thank the reviewer for taking the time to read and comment on our manuscript. We respectfully disagree that our study is fundamentally flawed, and provide detailed explanations below to address each concern.

The authors include the virus negative saliva samples in their transmission analysis; however, doing so convolutes the interpretation. When performing infection studies in mosquitoes there are two pieces of data that can be collected; how many individuals become infected (prevalence) and what is the burden of infection in those that became infected (intensity) (in this specific scenario virus in saliva and salivary glands). These are two fundamentally different questions and, therefore, should be separated in the analysis in my opinion. Looking at the data presented in Fig 1, it appears that NAK and CSP have lower levels of virus in the saliva than the DMSC strain but upon removal of the negative saliva samples this difference, especially for CSP, most likely disappears. Because so few had infection in the NAK strain it is difficult to determine if there would be a difference. Regardless, does the titer in the saliva actually matter? A previous study found that the human ID50 <10 pfu for dengue virus vaccine candidates (Blaney  doi: [10.1007/978-3-642-02215-9_11](https://doi.org/10.1007/978-3-642-02215-9_11)). To me, the more important metric is the prevalence of ZIKV in the saliva.

**Response:** We agree with the reviewer that both infection prevalence and infection intensity provide different insights into infection kinetics at each barrier. We also concur that prevalence is the most important parameter for assessing transmission potential, which is why we presented prevalence data separately in Figure 1A. In the revised version of the manuscript, we performed statistical comparisons and included 95% confidence intervals of the infection prevalence between groups to better demonstrate the difference among mosquito strains.

With regard to infection intensity, we believe this parameter remains crucial, as it may help to elucidate the biological basis of transmission barriers. Initially, we did not exclude uninfected mosquitoes from the intensity analysis because the low number of ZIKV-positive saliva samples limited the statistical power for group comparisons. After considering the reviewer’s comment, we have revised Figure 1 to exclude uninfected samples from the comparison of infection intensity, thereby focusing specifically on the viral burden in infected individuals. Moreover, we consider infection intensity in saliva biologically relevant, since female mosquitoes may engage in interrupted blood feeding, and a higher amount/concentration of infectious virus in saliva could increase the likelihood of transmission during such brief feeding events.

With that said, there are problems with the approach for assaying transmission, specifically the use of capillary tubes. A recent study (Gloria-Soria 2022 “Saliva collection via capillary method may underestimate arboviral transmission by mosquitoes”) demonstrated that “the methods of detecting virus infection in mosquito legs and in SG were equally accurate in predicting transmission. Overall, the presence of virus in mosquito legs was a more accurate predictor of transmission than the commonly implemented viral detection method using forced salivation into a capillary tube, and was subject to less technical variation.”

**Response:** We thank the reviewer for highlighting this important point, however we suggest that the interpretation of the results in the Gloria-Soria 2022 paper may be questionable due to certain technical aspects and the way the results have been interpreted. In the cited study, the transmission level measured with suckling mice was exceptionally high (close to 100% in most experiments) because the mosquitoes were infected by intrathoracic injection, which bypasses the midgut barrier. Therefore, it does not come as a surprise that legs and salivary gland infection (reaching 100% in all experiments due to the intrathoracic injection) is a better estimate for transmission in that study. In fact, discrepancies appear between legs/salivary gland infection and mouse infection in the experiments with lower mouse infection rates (such as Figure 1B in that paper). Thus, it can also be argued that the leg/salivary glands infection may overestimate transmission potential in certain biological settings. Furthermore, infection kinetics are temporal, and biologically a leg infection could be a misleading proxy when the infection has not yet reached the salivary glands, making transmission impossible.

We do understand that the forced salivation method may underestimate the transmission potential due to:

i) the sensitivity of the detection to detect low virus titers in the samples. We noticed that the saliva virus titers in Gloria-Soria 2022 were unusually low, which might be due to the fact that the study used only 2 µL of RNA samples from 50 µL of RNA (only 4% of total samples). **Instead, our study used at least 80% of the saliva sample for plaque assay or 20% of saliva sample for qPCR (see the schematic diagram in the Figure 4) thus having higher sensitivity for the detection.** Thereby reducing the chance of underestimation with the forced salivation.

ii) the amount of saliva collected from samples may be low and potentially vary among samples; however, **we subsequently verified in the Figure 3 that the observed differences were not merely due to variations in saliva volume.**

While there are additional points that could be discussed, we believe the explanations provided here already highlight valid limitations in the interpretation of the cited study.

Further, what is being collected during forced salivation may not accurately represent what is being salivated during blood feeding. Marinotti et al (“Diet and salivation in female Aedes aegypti mosquitoes”) found that Aae contain alpha-glucosidase in their proximal lobes and apyrase in their distal lobes and when fed a liquid sugar meal, levels of alpha-glucosidase decrease but not that of apyrase while the activity of both enzymes were reduced upon a blood meal. This suggests that the fundamental mechanisms mediating salivation may be different between forced salivation and actual blood feeding.

**Response:** We do agree that the saliva content during blood feeding and sugar feeding might be differ due to the sensing of different component in the food source. However, our study used cell culture medium supplemented with 10% fetal bovine serum, which more closely mimics blood feeding than sugar feeding.

Together, this suggests that the saliva data may not be a good metric to use when assessing transmission potential and that the salivary gland infection data may be better to identify differences between the populations; however, there were no differences at 14 and 21 dpi. Consequently, the premise for these studies and resultant conclusion about salivary gland escape barriers are flawed.

**Response:** We agree that forced salivation has limitations as a measure of transmission potential. Indeed, it is not a perfect proxy, and we acknowledge that alternative methods, such as using suckling mice, could provide additional information; however, these models are not available to all laboratories and raise ethical concerns. Importantly, in our study, we controlled experimental conditions carefully—mosquitoes were reared under the same conditions, infected with the same virus batch, fed with the same blood source, and all reagents were from the same stocks—so the observed variations are likely to reflect true biological differences. Moreover, we employed several complementary techniques to ensure that the observed differences in transmission potential were not due to technical variation. If we had only analyzed salivary gland infection data, we would not have identified the salivary gland infection barrier, which is a key finding of this study.

Figure 1: the authors state that “data was summarized from three blood feeding experiments with at least 10 mosquitoes from each”; however, when examining the prevalence data I see only two which I assume represent the replicate experiments. If this assumption is accurate shouldn’t there be three data points for the prevalence data?

**Response:** We thank the reviewer for pointing out this error. We confirm that the data presented in Figure 1 were summarized from two blood-feeding experiments, not three as previously stated. The manuscript has been updated to correct this typo.

Figure 1: It would be nice to include sample size values to the prevalence data in the figure as was done in Fig. 6B.

**Response:** All groups included 12 mosquitoes for the prevalence data, and we have updated the figure legend to reflect this. Following the reviewer’s suggestion, we think that including sample sizes in the revised infection intensity graph is beneficial, as the number of virus-positive samples can differ from the total number of mosquitoes. Accordingly, Figure 1B has been updated to display these sample sizes.

Reviewer #2: General Comment: This study evaluated a salivary gland escape barrier in several populations of Ae. aegypti. The presence of this genetically inherited barrier to transmission may be incorporated into disease prevention strategies. I am glad to see that the authors used virus detection rather than RT-PCR to detect ZIKV. The mere detection of viral RNA does not mean that any infectious virus was present. Similarly, I am glad to see that the authors used “days post-infectious blood meal” rather than the more commonly and incorrectly used “days post infection.” If it was days post infection, then all of the mosquitoes would have been infected. Overall, this is a very informative study and involved a lot of well-designed work. Below are several suggested changes. Most are merely style/format corrections.
**Response:** We thank the reviewer for their positive and constructive feedback, and we are grateful for their assessment of the study as informative and well-designed. We also appreciate the reviewer for taking time to explain language nuances and point out typos. All suggested changes have been carefully considered and are addressed below.

Specific Comments:

1. Lines 81-82: Why establish WNV, JEV, and YFV if they are not used again?

**Response:**  We have revised the Introduction to remove references to WNV, JEV, and YFV, as they were not used in this study.

2. Line 86: As this is the first time that Aedes aegypti is mentioned in the body of the paper, shouldn’t it be written out?
**Response:** We have revised the text to write out *Aedes aegypti* in full.

3. Line 100: Should “This immunity alone…” be “Thus, immunity alone…?”

**Response:** We have revised the text to “Thus, immunity alone”.

4. Line 114: Shouldn’t “human” be “humans” as it is referring to more than a single person. More importantly, as this is referring to SGEB in general, shouldn’t this be “vertebrate hosts…?”

**Reponse:** We do agree that “vertebrate hosts” is more accurate for the context. We have revised the text to reflect this as suggested.

5. Line 115: This should be in the past tense, so it should be “we investigated the…” Similarly, on line 117, “explore” should be “explored…” and on line 120. “we aim to…” should have been “we aimed to…”

**Reponse:** We have revised this paragraph to the past tense.

6. Line 255: Should “the transmission potential NAK population” be “the lower transmission potential NAK population?”

**Reponse:** We have corrected the typo to “the lower transmission potential NAK population?”.

7. Line 260: As “fifty” is not the first word in the sentence and is greater than 10, it should be “50 males…”

**Reponse:** The text has been revised as suggested.

8. Lines 331-335: Unless they are the first word in a sentence, numbers of measurement should not be written out and numerals should be used, e.g., 3 hours or 3 days. Note, this was correctly done on line 362 and others.

**Reponse:** We appreciate the reviewer taking the time to provide detailed feedback, which will help us improve not only this manuscript but also future work. The text has been revised as suggested.

9. Line 351: As ZIKV was established on line 80, there is no reason to reestablished here.

**Reponse:** The text has been revised as suggested.

10. Lines372-377: Yes, looking at Figure 1, I can see that the ability of the NAK strain to transmit ZIKV was significantly lower than that of either of the other two populations. You might want to modify the sentence to, “…varied significantly with transmission rates for the NAK being significantly (p < 0.0001) lower than that for the DMSC (Figure 1).”

**Reponse:** We agree that the NAK strain exhibited significantly lower transmission potential than the other two populations. Following revisions to Figure 1 and the text to address Reviewer 1’s concern, uninfected samples were removed from the saliva infection intensity analysis to separate comparisons of infection prevalence from infection intensity. In this analysis, we were able to estimate the differences in virus titers (effect size), in addition to statistical significance. Therefore, we included such information on the biological significance and statistical significance into the text.

Accordingly, we revised the text to: “In addition to differences in the prevalence of saliva infection, the quantity of infectious virus in ZIKV-positive samples varied consistently among populations (Figure 1B), with DMSC having the highest while the NAK having the lowest transmission potential. The infectious titers in the ZIKV-positive saliva of CSP were 0.76–1.51 log10 PFU lower, whereas those of NAK were 1.28–1.97 log10 PFU lower than those of DMSC (Figure 1B, effect size). These findings indicate that while all tested Ae. aegypti populations were susceptible to ZIKV infection and allowed systemic viral dissemination, their ability to transmit the virus varied significantly, with NAK showing the least transmission potential.” (Line 381-390 in the tracked changes version).

11. Line 474-475: Why reestablish dpibm as it was established on line 362.

**Reponse:** The text has been revised to dpibm without reestablishing the abbreviation.

12. Lines 489-490: While it is easy to calculate, viral titers to a hundredth of a log are usually not accurate and claim a degree of accuracy not supported by data. Therefore, I suggest that you change these to 5.5-5.6 log and 5.9-6.1 log…

**Reponse:** The texts have been revised as suggested.

13. Lines 590-591: Yes, the Aaa are more likely to feed on humans than the Aaf, and thus, are much more likely to be involved in the transmission of anthropomorphic viruses such as dengue and chikungunya. See the recent studies by Agha et al. Entomological assessment of dengue virus transmission risk in three urban areas of Kenya. PLoS Negl Trop Dis. 2019 Aug 23;13(8):e0007686 and Agha et al. Vector competence of populations of Aedes aegypti from three distinct cities in Kenya for chikungunya virus. PLoS Negl Trop Dis. 2017 Aug 18;11(8):e0005860. Not only is transmission efficiency critical, but feeding preference is also important

**Response:** We agree that both the vector competence and the host feeding preferences are important for the epidemiology of arbovirus transmission. We have revised the text to reflect this, as follows:

“Indeed, recent studies demonstrated that the relative proportion in genetic ancestry between two different forms of *Ae. aegypti* – *Ae. aegypti aegypti* (*Aaa*) and *Ae. aegypti formosus* (*Aaf*) – **influences both vector competence and host feeding preference, thus** shaping arbovirus transmission dynamics in Africa **[11,29,30].”** (Lines 624-625 in the tracked changes version)

14. References: These need to be formatted properly.
a. Only the first word and proper nouns in a reference title should be capitalized. See references 5, 15, 24 and many others.
b. Genus and species names should be in italics. See references 2, 3 4, and others.
c. Why is the name of the editor of some of the PLoS journal papers included? See references 5, 12 and others.
d. Shouldn’t PLOS ONE or PLoS ONE be PLoS One? See references 5, 12, and others? You need to be consistent.
e. Why is the title of reference 28 in all caps?

**Reponse:** We thank the reviewer for pointing out these issues. All references have been revised to ensure proper formatting.

Reviewer #3: To the Authors:

The manuscript entitled: “Natural salivary gland barrier curtails Zika virus transmission in Thai Aedes aegypti” by Sartsanga et al. is well written and clear. The study reports the discovery of a heritable salivary gland barrier, which may be used to form the basis of an alternative arbovirus control strategy using population replacement strategies as well as a tool to explore mosquito arbovirus interactions. Nice work.

**Response:** We thank the reviewer for their positive and encouraging comments.
